# On a sugar high: Role of O-GlcNAcylation in cancer

**DOI:** 10.1016/j.jbc.2023.105344

**Published:** 2023-10-12

**Authors:** Giang Le Minh, Emily M. Esquea, Riley G. Young, Jessie Huang, Mauricio J. Reginato

**Affiliations:** 1 Department of Biochemistry and Molecular Biology, Drexel University College of Medicine, Philadelphia, Pennsylvania, USA; 2 Translational Cellular Oncology Program, Sidney Kimmel Cancer Center, Thomas Jefferson University, Philadelphia, Pennsylvania, USA

**Keywords:** O-GlcNAc, OGT, hexosamine, cancer, metabolism, metastasis

## Abstract

Recent advances in the understanding of the molecular mechanisms underlying cancer progression have led to the development of novel therapeutic targeting strategies. Aberrant glycosylation patterns and their implication in cancer have gained increasing attention as potential targets due to the critical role of glycosylation in regulating tumor-specific pathways that contribute to cancer cell survival, proliferation, and progression. A special type of glycosylation that has been gaining momentum in cancer research is the modification of nuclear, cytoplasmic, and mitochondrial proteins, termed O-GlcNAcylation. This protein modification is catalyzed by an enzyme called O-GlcNAc transferase (OGT), which uses the final product of the Hexosamine Biosynthetic Pathway (HBP) to connect altered nutrient availability to changes in cellular signaling that contribute to multiple aspects of tumor progression. Both O-GlcNAc and its enzyme OGT are highly elevated in cancer and fulfill the crucial role in regulating many hallmarks of cancer. In this review, we present and discuss the latest findings elucidating the involvement of OGT and O-GlcNAc in cancer.

Cancer is still a major health challenge worldwide accounting for nearly 10 million deaths in 2020 (1, 2). The development of cancer is caused by accumulations of various mutations induced by exposure to chemicals or agents that cause DNA damage which leads to alteration in cancer cell metabolism, cell survival, proliferation, signal transduction, and ultimately results in uncontrolled growth (2, 3). The uncontrolled proliferation of cancer cells imposes a heavy burden on primary organs leading to organ failure and even mortality. Furthermore, cancer cells also possess the capability to spread and metastasize to distant tissues and organs, which is often associated with worsened clinical outcomes and an increase in death rate in patients with cancer (2).

Since the past decades, cancer metabolism has been extensively studied and recognized as a signature hallmark of tumor progression (4). The metabolic phenotypes of cancer cells are distinct from normal cells as cancer cells prefer glycolysis over oxidative phosphorylation to support the requirement in generating biomass needed for rapidly proliferating cells (5, 6). However, recent studies have revealed that metabolism of cancer cells is highly dynamic, which means that cancer cells are capable of adjusting their metabolism to adapt to the varying microenvironments, including during metastasis. This characteristic of cancer metabolism can be referred to as metabolic plasticity (7, 8). Metabolic plasticity of cancer cells is also associated with high plasticity observed in signal transduction pathways, which is heavily influenced by genetic alterations as well as by stimuli from the tumor microenvironment. Consequently, understanding the mechanism by which cancer cells sense the nutritional status and couple alteration in tumor microenvironment to signaling, metabolism, and other critical cellular activities of the cancer cells, has emerged as a major area of cancer research (9).

One nutrient-sensing mechanism that is frequently altered in cancer and has recently received increasing attention is the hexosamine biosynthetic pathway (HBP). The HBP utilizes products from major metabolic pathways, including glucose (from carbohydrate metabolism), glutamine (from protein and amino acid metabolism), acetyl-CoA (from lipid and fatty acid metabolism), and uridine triphosphate (UTP) (from nucleic acid and nucleotide metabolism) to form a nucleotide sugar UDP-N-acetylglucosamine (UDP-GlcNAc) (10) (Fig. 1). This nucleotide sugar serves as the substrate for glycosylation of membrane and secreted proteins that perform a vital role in signaling transduction as well as in extracellular matrix formation. The critical role of glycosylation in regulating the functional activities of these extracellular proteins has been extensively discussed in other reviews (11). In addition to glycosylation of membrane and secreted proteins, intracellular proteins, including nuclear-cytoplasmic and mitochondrial proteins, can also be glycosylated in a process called O-GlcNAcylation (10). This modification, which occurs at serine and threonine residues of target proteins, analogous to phosphorylation, regulates protein stability, localization, and the protein phosphorylation state (10). However, unlike phosphorylation, O-GlcNAcylation is facilitated by a single ubiquitously expressed enzyme called O-GlcNAc transferase (OGT). O-GlcNAcylation is a reversible modification and can be removed by another ubiquitously expressed enzyme called O-GlcNAcase (OGA) (12). Dynamic activities of OGT and OGA result in homeostasis of O-GlcNAcylation, dysregulation of which is often associated with various pathological conditions (13). Decreased expression of OGT and lowered O-GlcNAc levels have been found to be linked to certain cognitive disorders (14), while elevated expression of OGT and increased O-GlcNAc levels are often associated with metabolic diseases including cancer (10). In cancer cells, OGT and O-GlcNAc couple alteration in nutrient status to signaling activities, which contributes to reprogramming cellular metabolism, as well as to modification of transcriptomic and proteomic profiles, driving the progression of cancer (15).

**Figure 1:**
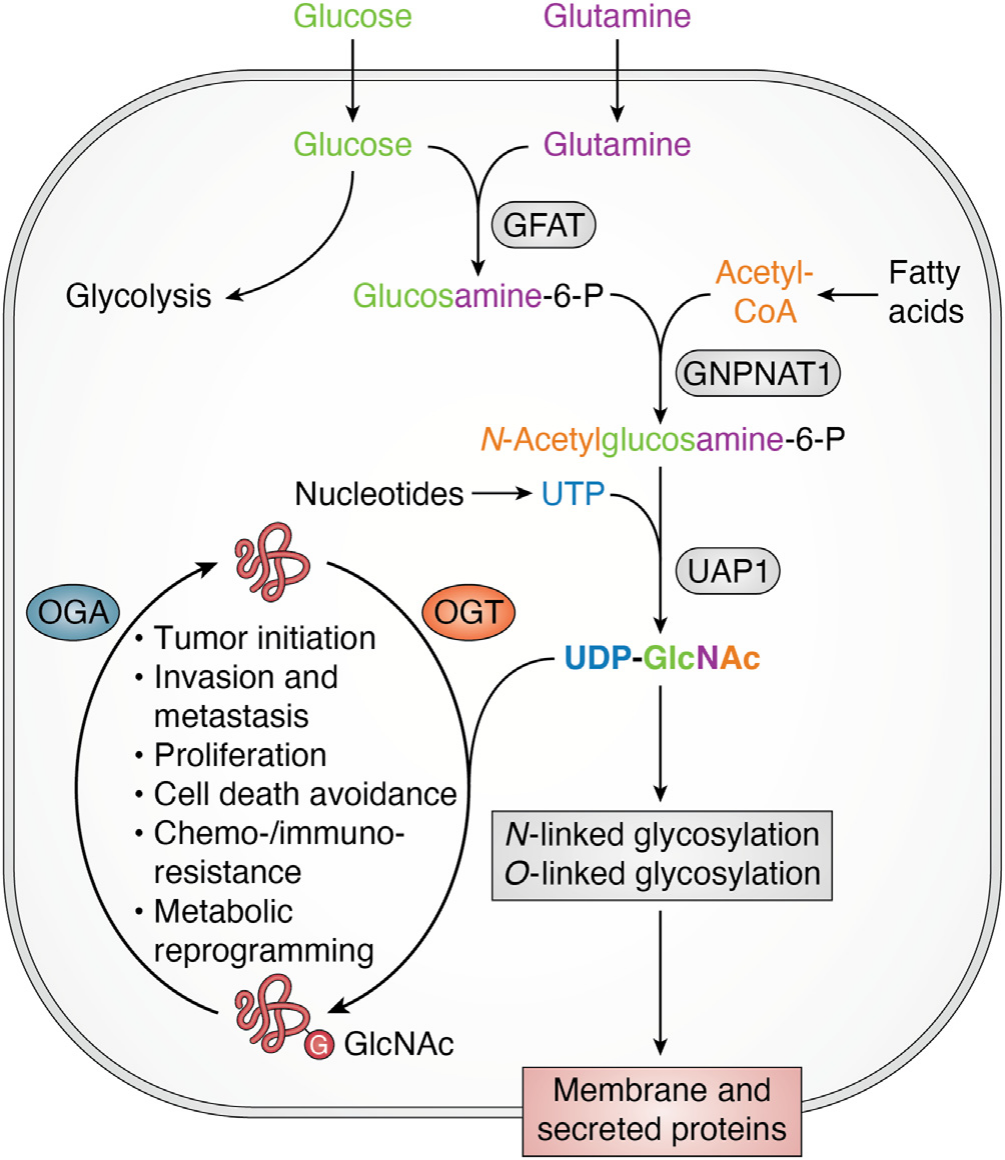
**Hexosamine biosynthetic pathway and O-GlcNAcylation cycle connect nutrient status to cancer cellular activities.** Major products from different metabolic pathways are used in the HBP to generate UDP-GlcNAc as a sensor for nutrient status of the cells. UDP-GlcNAc is used by OGT to modify proteins and is removed by OGA, resulting in O-GlcNAcylation cycle. Elevated OGT and O-GlcNAc levels are associated with increased proliferation, survival, chemoresistance, and metastasis of cancer cells. Created with BioRender.com.

In recent years, there has been a growing understanding of how OGT and O-GlcNAc regulate cancer cell proliferation and survival, metastasis, drug resistance, and other cancer-associated phenotypes. Here in this review, we will present and discuss the latest findings that shed light on the involvement of OGT and O-GlcNAc in tumor development. Furthermore, we will discuss the potential and challenges in targeting OGT and O-GlcNAc as an approach to combat cancer.

## The hexosamine biosynthetic pathway

The hexosamine biosynthesis pathway (HBP) is a branch of glucose metabolism, in which a small percentage of glucose entering the cell (3–5%) is shunted into a pathway that utilizes glucose together with other metabolic products to form the final product of UDP-GlcNAc (10). The HBP and its final product have been reported to be critical for embryonic development (16). Pharmacological or genetical inhibition of the HBP delays embryonic development (17), while elevated activity of the HBP is associated with increased resistance to insulin (17, 18). Similar to other nutrient sensing mechanisms, such as the AMP-activated protein kinase (AMPK) pathway and mammalian target of rapamycin (mTOR) pathway, dysregulation of the HBP is strongly associated with cancer. The activity of the HBP and the level of UDP-GlcNAc are directly influenced by alterations in nutrient status within the tumor microenvironment. Therefore, the HBP plays a crucial role in coupling these changes to the regulation of signaling pathways in cancer cells (10, 15).

The activity of HBP and the level of UDP-GlcNAc are often increased in cancer when compared to normal tissues (19). This increase in HBP activity and UDP-GlcNAc level could be a result of an increased influx of metabolites such as glucose and glutamine into the HBP pathway (20). However, other reports also suggested that the expression of key enzymes of the HBP is elevated in cancer, which might result in the elevated activity of the HBP and potentially contribute to drive tumor growth (21, 22, 23, 24, 25, 26, 27, 28).

The rate-limiting enzyme of the HBP is glutamine fructose-6-phosphate amidotransferase (GFAT), which catalyzes the reaction between fructose-6-phosphate derived from glucose entering the cells, and glutamine, to form glucosamine-6-phosphate (29). Elevated expression of GFAT has been observed in breast cancer, which is closely correlated with an increase in UDP-GlcNAc level (30). In non-small cell lung cancer, the expression of GFAT is higher compared to normal tissue (31). This increase in GFAT expression promotes the proliferation of lung cancer cells. Importantly, inhibition of GFAT sensitizes cancer cells to *in vitro* treatment with cisplatin, a DNA-damage-inducing drug that is widely applied to treat cancer (31, 32). These findings not only point to the role of GFAT in driving tumor growth but also suggest a potential synergistic effect between targeting HBP enzymes and anti-cancer chemotherapy.

The enzyme glucosamine-6-phosphate N-acetyltransferase (GNPNAT1), which incorporates acetyl-CoA into the HBP, is also overexpressed in multiple cancers (29). High expression of GNPNAT1 was reported in lung cancer and was associated with poor survival rate of patients with lung cancer (25). This increase in expression of GNPNAT1 also has potential prognostic value as a marker for lung adenocarcinoma (25, 33). In contrast, reduced expression of GNPNAT1 in castration-resistant prostate cancer cells significantly increased cancer cell proliferation and tumorigenic capacity (34). These reports may indicate a cancer-specific role of GNPNAT1 in maintaining the homeostasis of cancer cells during the process of tumor development.

The final step of the HBP is catalyzed by UDP-N-acetylglucosamine pyrophosphorylase 1 (UAP1), which generates UDP-GlcNAc from substrates of N-acetylglucosamine-1-phosphate and UTP (29). UAP1 expression is highly elevated in prostate cancer, where the increase in expression correlates with poor survival rate of patients with prostate cancer. Inhibiting expression of UAP1 sensitized prostate cancer cells to the inhibitor of N-linked glycosylation (23). Elevated expression of UAP1 is also reported in bladder cancer, where its expression is critical for bladder cancer cell proliferation, migration, and invasion (35). These findings point to the crucial role of UAP1 in promoting cell survival and proliferation in various cancers, and its potential as a target for anticancer therapies.

Correlated with the increase in HBP activity and the overexpression of HBP enzymes, elevated level of UDP-GlcNAc has also been observed in multiple cancers. In breast cancer, accumulation of UDP-GlcNAc correlates with the biosynthesis of hyaluronan, a marker for disease progression and poor prognosis (30). Similarly, an increased level of UDP-GlcNAc is observed in prostate cancer (23). In lung cancer cells, increases in HBP activity and UDP-GlcNAc production are directly associated with epithelial–mesenchymal transition (EMT), a critical process for cancer cell migration and invasion (36).

UDP-GlcNAc can be used for glycosylation of membrane and secreted proteins in the ER and Golgi apparatus. In this process, UDP-GlcNAc can be incorporated directly into the sugar chain of glycosylated proteins, or it can serve as a precursor for generating other nucleotide sugars needed for N-lined and O-linked glycosylation, such as UDP-N-acetylgalactosamine and CMP-N-acetylneuraminic acid (10). The connection between HBP and glycosylated membrane and secreted proteins in cancer has been extensively discussed elsewhere (20). The remaining protein modification that utilizes UDP-GlcNAc, O-GlcNAcylation, and its involvement in cancer will be discussed extensively in the following sections.

## Contributions of OGT/O-GlcNAcylation in cancer

The O-GlcNAc cycling process is controlled by two ubiquitously expressed enzymes, OGT and OGA, which regulate the function of a wide range of intracellular proteins (10, 15). This dynamic regulation by O-GlcNAc results in a variety of outcomes on the target proteins. One key factor that contributes to the effect of O-GlcNAc modification is the protein interaction profile exhibited by OGT and OGA. However, because OGT and OGA are highly conserved with a very limited number of variants observed in human cells, this divergence in protein interaction profiles of both OGT and OGA is largely attributed to the structural properties of these enzymes (13).

OGT is essential for embryonic development in mammals (37). Since its first discovery in 1990 (38, 39), significant progress has been made in determining the structure of OGT. Structural analysis of the enzyme shows a catalytic domain at the C-terminus, which is split into N-catalytic and C-catalytic half-domain by an intervening (Int-D) region. At the N-terminus of OGT, there is a domain containing multiple tetratricopeptide repeats (TPR), which is required for interaction with other binding partners. The number of these repeats varies between isoforms of OGT. The main isoform, nuclear-cytoplasmic OGT (ncOGT), contains 13.5 TPRs, while mitochondrial OGT (mOGT) contains nine TPRs. The TPR domain serves as a scaffold to facilitate protein-protein interaction and also functions as a regulatory site for controlling the interaction of OGT with other proteins, as well as for OGT catalytic and non-catalytic activities (13). Both catalytic and non-catalytic functions of OGT are required for mammalian cell proliferation and are associated with cancer-related phenotypes (10, 13, 15, 40).

The remaining enzyme of O-GlcNAc cycling, OGA, is also expressed in all human tissues and is also essential for embryonic development. Genetic depletion of OGA results in perinatal lethality and development delay (41). OGA removes the O-GlcNAc moiety from target proteins in the cell nucleus and cytoplasm and is distinct from other hexosaminidases found in lysosomes (13). OGA contains three different domains in its structure. The catalytic domain is found at the N-terminus, which is linked to the pseudo histone acetyltransferase (pHAT) domain at the C-terminus *via* a stalk domain. The intrinsic disorder nature of this stalk domain is believed to be critical for protein-protein interaction between OGA and its binding partners (42). On the other hand, the pHAT domain is highly conserved in mammals and is proposed to be critical for the selectivity and specificity of OGA (43).

Together, the combined activities of OGT and OGA create a dynamic cycling of O-GlcNAcylation in mammalian cells to maintain cellular homeostasis. In cancer cells, this homeostasis is altered, and the level of O-GlcNAcylation is elevated in nearly all cancers examined (10, 13, 15, 40). This increase in O-GlcNAcylation level, together with increased OGT expression, promotes cancer cell proliferation, survival, chemoresistance, stem-like cell phenotypes, invasion, and metastasis (10, 13, 15, 20, 29, 40) (Fig. 1). In this section, we will extensively discuss the latest findings on the role of OGT and O-GlcNAc in cancer.

### Role of OGT in cancer cell metabolism

Cancer cells differ from normal cells in their ability to proliferate and survive, but also in their metabolism. During tumor development, the metabolism of cancer cells is drastically reprogrammed to meet the demand for rapid proliferation and is regulated by various internal and external signals. The alteration in cancer cell metabolism is influenced by the availability of nutrients in the tumor microenvironment, which is detected by nutrient-sensing mechanisms, such as OGT and O-GlcNAc (8). OGT and O-GlcNAc dynamically couple alterations changes in the nutrient’s status to cellular metabolism, thereby modulating a diverse range of cell signaling pathways (15). These alterations in cell signaling, in turn, have a significant impact on the reprogramming of metabolic pathways, including glycolysis/oxidative phosphorylation, lipid metabolism, amino acid metabolism, and others (44).

#### OGT/O-GlcNAc regulates key enzymes of glycolysis and the TCA cycle

**Figure 2:**
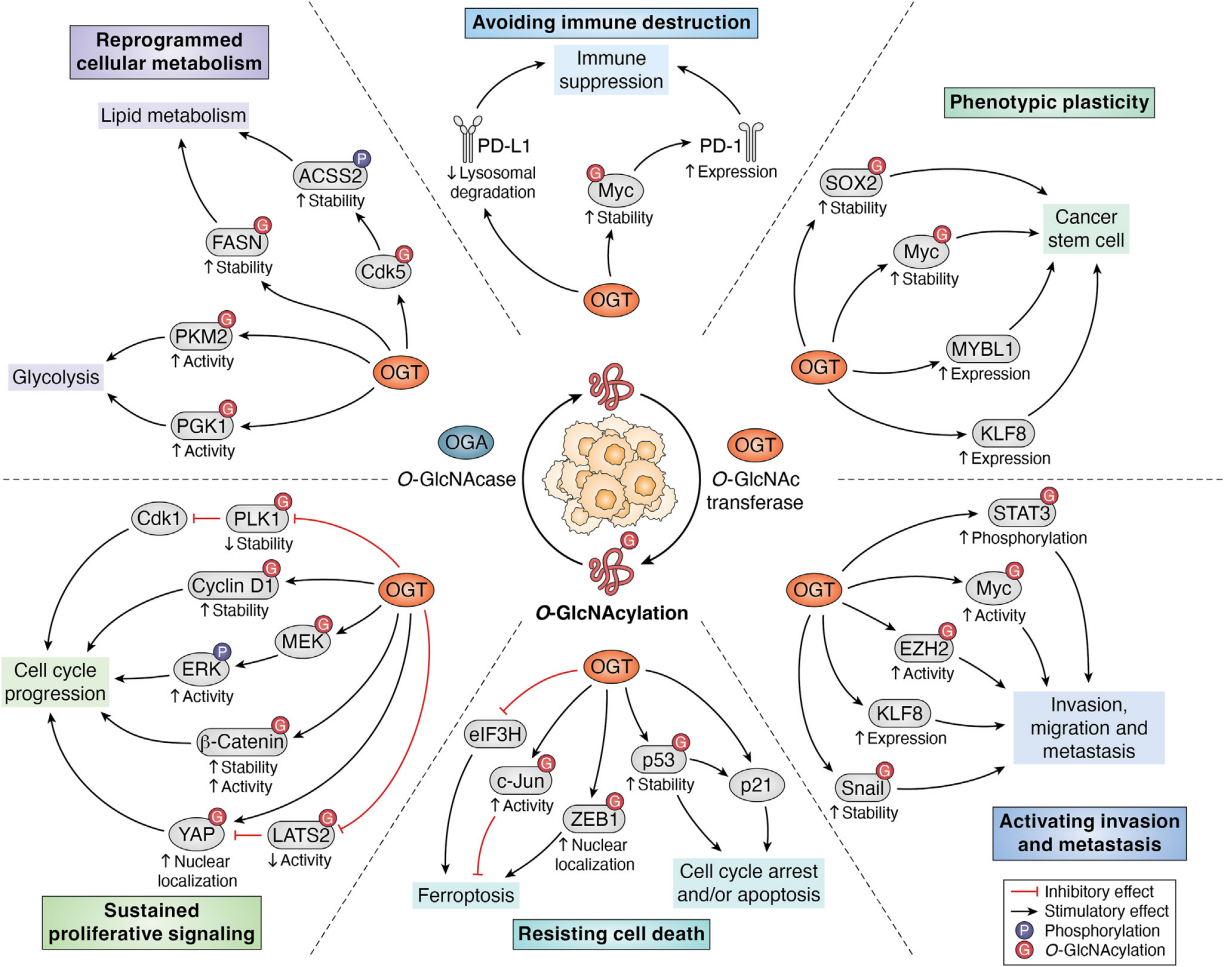
**OGT/O-GlcNAc regulates major hallmarks of tumor progression.** OGT and O-GlcNAc are critical in regulating metabolic profile and significantly contribute to driving metabolic plasticity of cancer cells. OGT and O-GlcNAc are also involved in promoting cancer cell proliferation and survival, contributing to driving resistance to anti-cancer therapies. OGT and O-GlcNAc in many cancers are critical for metastasis by regulating invasion/migration and cancer stem-like cell properties. Some newly discovered mechanisms of cancer cell activity are listed. Created with BioRender.com.

Cancer cell metabolism is reprogrammed to preferentially utilize glycolysis over oxidative phosphorylation to meet the requirements for tumor growth (8). Inhibitions of OGT and O-GlcNAc lead to reduced glycolysis and survival in breast cancer cells (45), suggesting a potential regulation of glycolysis by OGT and O-GlcNAc. Indeed, many glycolytic enzymes are directly modified with O-GlcNAcylation. Phosphoglycerate kinase 1 (PGK1), an essential enzyme of glycolysis, is highly expressed in colon cancer and is associated with increased cell proliferation (46, 47), as well as with resistance to chemoradiotherapy (48) and overall poor patient prognosis (48). PGK1 is O-GlcNAcylated at threonine residue 255, which activates PGK1 activity and induces translocation of PGK1 to into the mitochondria, enhancing glycolysis (49). Conversely, blocking O-GlcNAcylation of PGK1 decreases cell proliferation and suppresses glycolysis in colon cancer cells (49). Another key enzyme in glycolysis, the pyruvate kinase M2 (PKM2) is a target of OGT and O-GlcNAc. O-GlcNAcylation of PKM2 at threonine residue 405 and serine 406 enhances PKM2 oligomerization and PKM2 enzymatic activity increasing glycolytic activity (50, 51). In addition, PKM2 regulation by OGT is augmented by epidermal growth factor (EGF), which is highly expressed in various cancers to promote cell survival and proliferation (52). Activation of EGF signaling leads to phosphorylation of OGT at tyrosine residue 976, which in turn augments the interaction between OGT and PKM2 to upregulate glycolysis. These studies stress the importance of OGT in promoting glycolytic cancer phenotypes (Fig. 2).

Recent studies have shown that mitochondria also play a central role in regulating the plasticity of cancer cell metabolism (8). Over the last decade, several groups have investigated the connection between O-GlcNAc and mitochondria function (53, 54) as well as the role of mitochondrial OGT, or mOGT (55, 56). Overexpression of OGT or OGA showed a significant decrease in the level of mitochondria-localized proteins involved in the electron transport chain and the TCA cycle (53). In a cardiac myocyte study, hyper O-GlcNAcylation increased maximum respiratory capacity and decreased ROS (57), suggesting that OGT/O-GlcNAcylation could play a similar role in cancer. Indeed, recent proteomic studies in breast cancer cells have shown that mOGT can interact and modify many proteins that regulate mitochondrial respiration, fatty acid transport, and metabolism (55, 56, 58). However, the contribution of mOGT in regulating mitochondrial metabolism in cancer cells still needs to be further evaluated.

#### OGT/O-GlcNAc regulates lipid metabolism

In addition to reprograming carbohydrate metabolism, cancer cells also alter their own lipid metabolism, where the cell utilizes *de novo* lipid synthesis to generate fatty acids (59, 60). O-GlcNAc has been shown to regulate lipid metabolism in various cancers including breast (61), brain (62), liver (60), and melanoma (63). Global metabolomic analysis using liquid chromatography-mass spectrometry has been used to examine the impact of O-GlcNAc on cancer metabolism. Cancer cells with reduced OGT level showed significant reduction in lipid metabolites and lipid-synthesis-associated metabolites (61). Breast cancer cells depleted of OGT also showed a reduction in sterol regulatory element binding protein 1 (SREBP-1) expression. SREBP-1 is the master regulator of lipid homeostasis and is required for the synthesis of fatty acid, cholesterol, and phospholipid (64). While SREBP-1 is not directly modified by OGT, depletion of OGT leads to an increase in the interaction between SREBP-1 and its E3-ubiquitin ligase FBW7 suggesting that OGT and O-GlcNAc indirectly regulate the stability of SREBP-1. In addition, this study by Sodi. et.al. also showed that under metabolic stress like OGT depletion, the AMPK pathway is activated leading to an increase in phosphorylation of SREBP-1, which ultimately reduces the level of total SREBP-1 and the level of its transcriptional target Fatty Acid Synthase (FASN) (61). Taken together, this study demonstrated that OGT/O-GlcNAc regulates lipid metabolism in breast cancer cells, in part, by regulating the stability of SREBP-1 (61).

The process of *de novo* lipid biosynthesis is also controlled by OGT/O-GlcNAc in cancer (65). Knockdown of serine/arginine-rich protein-specific kinase 2 (SRPK2) resulted in an impairment of *de novo* lipogenesis similar to the phenotype observed in breast cancer cells with OGT depletion (65). SRPK2 is a protein kinase responsible for mediating pre-mRNA splicing (66), and is activated by mTORC1 and ribosomal S6 kinase (S6K1) to control the processing of mRNA of various lipogenic genes including FASN and ATP-Citrate Lyase (ACLY) (67). In breast cancer cells, SRPK2 is modified with O-GlcNAc at its nuclear localization sequence, which enhances the binding of SRPK2 to importins, thus increasing nuclear translocation of SRPK2, independent of SRPK2 mTORC1/S6K1 phosphorylation (65). These results also suggest lipid metabolism can also be regulated at the posttranscriptional level *via* OGT/O-GlcNAc in cancer cells.

A proteomics-based study of the O-GlcNAcome in HeLa cells confirmed a direct relationship between O-GlcNAcylation and *de novo* lipogenesis (68) *via* regulation of FASN, the rate-limiting enzyme in fatty acid synthesis (69). In these cells, FASN is O-GlcNAcylated, and an increase in O-GlcNAc levels results in enhanced *de novo* lipid synthesis. Importantly, dual inhibition of OGT and FASN synergized to induce the death of cancer cells *in vitro* (68). Similarly, FASN is found to be directly modified with O-GlcNAc in liver cancer, which protects the protein from proteasomal degradation and ultimately leads to an increase in lipogenesis (60). Together, these studies reveal the regulation of *de novo* lipid synthesis by OGT/O-GlcNAc in cancer cells (Fig. 2).

Another metabolic pathway in cancer that has been under increasing attention lately is acetate metabolism (70). Similar to lipid metabolism, acetate metabolism in cancer cells is also regulated by OGT/O-GlcNAc in glioblastoma (GBM) (62). Likely due to the high consumption of glucose by brain cells, brain tumors are heavily dependent on acetate (71) and require acetyl-CoA synthase 2 (ACSS2) to convert acetate into acetyl-CoA for *de novo* lipogenesis (72). The function of ACSS2 is heavily influenced by OGT and O-GlcNAc, which are highly expressed in glioblastoma (GBM) cells (62). In GBM cells, OGT promotes the phosphorylation of ACSS2 by increasing the activity of cyclin-dependent kinase 5 (CDK5). CDK5 phosphorylates ACSS2 at serine residue 267, which protects ACSS2 from proteasomal degradation and ultimately results in an increase in the conversion of acetate into acetyl-CoA and lipid accumulation (62). Importantly, the growth of intracranial tumors is impaired by OGT inhibition, which is successfully rescued by overexpressing phosphomimic ACSS2-S267D mutant, further implicating the crucial role of OGT and O-GlcNAc in promoting glioblastoma growth and survival *via* regulation of acetate and lipid metabolism (62).

#### OGT/O-GlcNAc regulates amino acid metabolism

The HBP utilizes amino acids to generate UDP-GlcNAc, which is subsequently used for O-GlcNAcylation. Therefore, the level of O-GlcNAc and the activity of OGT are tightly regulated by the availability of amino acids (73). In this manner, amino acid metabolism and O-GlcNAc are closely connected. The metabolism of glutamine, a substrate of the HBP, is tightly linked to the level of O-GlcNAc in cancer cells. Pancreatic cancer cells with deletion of glutamate ammonia ligase (GLUL), a key enzyme of glutamine synthesis, show a significant reduction in the total level of O-GlcNAc as well as a reduction in tumor growth (74). On the other hand, pharmacological inhibition of OGT blocks the growth of pancreatic cancer cells with intact GLUL expression and function, suggesting a critical role of OGT and O-GlcNAc in the regulation of cancer cell growth by glutamine metabolism (74). In colorectal cancer, elevated levels of OGT and O-GlcNAc increased glutamine uptake by increasing the expression of glutamine transporters SLC1A5 and SLC38A2 (75). In these cancer cells, the activity of OGT is controlled by cell migration-inducing and hyaluronan-binding protein (CEMIP), which functions as an adapter protein to facilitate O-GlcNAcylation of β-catenin, which in turn promotes nuclear localization of β-catenin (75, 76). Accumulation of β-catenin in the nucleus promotes the expression of glutamine transporters to enhance glutamine uptake in colorectal cancer cells in a CEMIP and OGT-dependent manner (75). In addition to glutamine, other amino acids in cancer cells are also regulated by OGT and O-GlcNAc. In prostate cancer, inhibition of OGT results in the depletion of intracellular alanine (77). Additionally, pharmacological inhibition of OGT increases the expression of alanine aminotransferase 2 (GPT2), which can be targeted to reduce cancer cell survival and proliferation (77). Although certain connections between OGT/O-GlcNAc expression and amino acid metabolism and their role in cancer have been explored, further investigation is required to generate a more comprehensive understanding of these mechanisms.

### Role of OGT in cancer cell proliferation and survival

#### OGT/O-GlcNAc regulates the cell cycle

In cancer cells, inhibition of OGT results in reduced cell proliferation. RNAi-induced inhibition of OGT leads to an accumulation of bladder cancer cells in G0/G1 phases, and a reduction of cells in the S and G2/M phases. OGT knockdown also reduces the expression of pro-proliferation protein cyclin D1, while increasing the level of cell cycle arresting protein p21 (78). Similarly, inhibition of OGT expression in hepatocarcinoma cells by miR-15-a results in cell cycle arrest and accumulation of cancer cells in G0/G1 phases, which ultimately leads to impaired proliferation of liver cancer cells (79). These findings highlight the direct involvement of OGT in cell cycle regulation. However, the detailed mechanism of cell cycle regulation by OGT and O-GlcNAc is still being explored. Recent studies have started showing the regulation by OGT on core components of the cell cycle. In breast cancer and colorectal cancer, a key regulator of the cell cycle, cyclin D1, is directly modified by OGT. This modification enhances cyclin D1 stability by protecting it from ubiquitination and degradation. Increased levels of cyclin D1 in cancer cells with high levels of OGT/O-GlcNAc results in an increase in cell proliferation (80). Another key regulator of the cell cycle that is regulated by OGT is cyclin-dependent kinase 1 (Cdk1). Cdk1 activity is inhibited by phosphorylation induced by Myelin transcription factor 1 (MYT1) and by the reduced expression of Cdk1 phosphatase called cell division cycle 25C (cdc25) (81), which is highly expressed in cancer and is associated with tumor development (82). Overexpression of OGT reduces both the mRNA and protein level of Polo-like kinase 1, an upstream regulator of both MYT1 and cdc25, thus OGT indirectly regulates the activity of Cdk1 during the cell cycle (81). These results highlight the complex role of OGT and O-GlcNAc in cell cycle regulation (Fig. 2) and indicate the need for further studies to understand the regulation of cell cycle kinases and related proteins by OGT and O-GlcNAc.

Inhibition of OGT also resulted in impaired DNA synthesis needed for cell division. In cervical cancer cells, OGT directly modifies the master kinase critical for DNA synthesis and chromosome segregation, PLK1 at Threonine residue 291 (83). This modification of PLK1 by OGT decreases the protein stability, and O-GlcNAc-deficient mutants of PLK1 show an increase in PLK1 level and an increase in chromosome segregation defects (83). This finding not only further confirms the role of OGT and O-GlcNAc in regulating the cell cycle but also implied the potential role of OGT and O-GlcNAc in maintaining genome stability in cancer cells.

#### Regulation of MEK/ERK signaling by OGT/O-GlcNAc

In addition to core regulators of the cell cycle, cancer cell proliferation is also heavily influenced by upstream signaling pathways. The MEK/ERK pathway is well-known to be hyperactivated in cancer and is often associated with increased proliferation and survival of cancer cells (84). The expression and activity of proteins involved in the MEK/ERK pathway have been reported to be modulated by OGT and O-GlcNAc in cancer. In breast cancer, MEK2 is directly modified by OGT at Threonine residue 13, which enhances MEK2 phosphorylation and ERK1/2 activation driving cancer cell proliferation (85). Similarly, breast cancer cells with inhibited OGT expression show a reduction in phosphorylation of ERK, which leads to a reduction in expression of pro-proliferation protein Forkhead Box M1 (FoxM1) (86, 87). In cholangiocarcinoma, increased O-GlcNAc level enhances tumor progression *via* activation of ERK signaling (88). These findings suggest that MEK/ERK signaling in cancer cells is partially regulated by OGT and O-GlcNAc. However, the interconnection between OGT/O-GlcNAc and MEK/ERK is not a one-way interaction. The level and function of OGT and O-GlcNAc in cancer cells are also modulated by the MEK/ERK pathway. In pancreatic cancer, ERK signaling plays a critical role in the maintenance of high OGT and O-GlcNAc levels, which is essential for cancer cell proliferation (89, 90). A similar regulation is also observed in esophageal squamous cell carcinoma, where ERK increases OGT expression driving cancer cell proliferation (91). Together, these findings reveal the dynamic interaction between OGT/O-GlcNAc and the MEK/ERK signaling and their role in regulating cancer cell proliferation and tumor progression.

#### OGT/O-GlcNAc regulates PI3K/Akt pathway in cancer

Similar to the regulation of the MEK/ERK pathway in cancer, the PI3K/Akt pathway, another major pathway in cancer, is also regulated by OGT and O-GlcNAc. Cancer cells with increased expression and activity of the PI3K/Akt pathway show an increase in cell proliferation and tumor growth (92). In addition, upregulation of the PI3K/Akt pathway is often observed in drug-resistant cancer cells, suggesting a potential role of the PI3K/Akt pathway in promoting cell survival under conditions of cellular stress (93, 94). In renal cancer cells, inhibition of OGT reduces expression of pro-proliferation and pro-survival protein EGF receptor (EGFR) as well as expression of its downstream targets PI3K/Akt. Decreases in the expression of EGFR and PI3K/Akt signaling result in delayed entry of cancer cells into S and G2/M phases, which ultimately reduces the proliferation of renal carcinoma cells (95, 96). In cervical cancer, a high level of O-GlcNAc is correlated with an increase in phosphorylation of Akt and is critical for the proliferation of cancer cells (97). In hypopharyngeal squamous carcinoma, a high level of OGT and O-GlcNAc enhances the stability NF-E2-related factor 2 (NRF2), which is closely associated with survival and chemoresistance of cancer cells, *via* activation of the PI3K/Akt pathway to drive the tumor growth (98, 99). These findings support the extensive crosstalk between OGT/O-GlcNAc and the PI3K/Akt signaling, as well as its importance during cancer progression.

#### OGT/O-GlcNAc regulates the mTOR signaling in cancer

A nutrient-sensing pathway that is tightly linked to the PI3K/Akt pathway and is frequently disrupted in cancer is mTOR signaling. Increased mTOR signaling promotes cancer cell proliferation, as well as survival (92). The activity of mTOR signaling in hepatocarcinoma cells is upregulated by OGT and O-GlcNAc to drive cancer cell proliferation. In these cancer cells, the level of O-GlcNAc correlates with the expression level of FASN, which is a positive regulator of mTOR signaling (60). FASN has been shown to be O-GlcNAcylated, which enhances the enzymatic activity of this fatty acid synthesizing enzyme, further supporting the intensive crosstalk between OGT, FASN, and the mTOR pathway in cancer (68). In colorectal cancer, OGT and O-GlcNAc activate the Akt/mTOR signaling pathway to control tumor progression. Genetic inhibition of OGT in colorectal cancer cells reduces phosphorylation of Akt and mTOR, thus impairing cancer cell proliferation. In contrast, overexpression of OGT increases phosphorylation and activity of Akt and mTOR, which in turn enhances the proliferation and metastasis ability of colorectal cancer cells (100). In breast cancer, silencing OGT expression reduces mTOR expression and impairs cancer cell proliferation (101). The mTOR signaling pathway also provides a feedback mechanism to regulate OGT and O-GlcNAc levels in breast cancer cells. Elevated mTOR activity in breast cancer cells enhances the expression of c-MYC to increase OGT level *via* upregulating HSP90A, which protects OGT from degradation by proteasome (102). Together, these findings reveal the extensive crosstalk between OGT and mTOR pathway and their role in regulating cancer cell proliferation and tumor growth.

#### OGT/O-GlcNAc regulates the Wnt/β-catenin signaling in cancer

The Wnt/β-catenin signaling pathway is another pathway that is crucial for cancer cell proliferation and survival. The Wnt/β-catenin is highly up-regulated in various cancers, where its elevated activity drives tumor progression (103, 104). Increased expression of Wnt/β-catenin is also associated with increased ability of cancer cells to avoid cell death caused by drug treatments (105). In cancer cells, β-catenin is directly modified by OGT at Threonine residue 41, which increases the stability of the protein contributing to drive cancer cell proliferation (76, 106). The direct O-GlcNAc modification on β-catenin also alters the protein-protein interaction profile of β-catenin, where it promotes the interaction between β-catenin and EZH2, which is a master regulator of the cell cycle and plays a pivotal role in driving tumor initiation, tumor growth, metastasis and drug resistance (107, 108). The interaction between EZH2 and β-catenin results in the recruitment of EZH2 to its target promoters to modulate gene expression in a Wnt/β-catenin dependent manner (107). Additionally, studies have also shown that OGT and components of the Wnt/β-catenin pathway cooperatively drive cancer cell tumorigenicity. In hepatoblastoma, O-GlcNAcylation of La-related protein (LARP1), which is an RNA-binding protein and is involved in various cancers, protects the protein from proteasome-dependent degradation. Increased LARP1 level enhances the expression of β-catenin increasing cancer cell proliferation and tumor progression (109). In colorectal cancer, pharmacological inhibition of OGT reduces the activity of β-catenin and reduces cell proliferation. This regulation of β-catenin by O-GlcNAc is mediated by phosphoglucomutase 3 (PGM3), which is highly expressed in colorectal cancer and is sufficient to elevate total level of O-GlcNAc in colorectal cancer cells (110). In addition to promoting cell proliferation, OGT and Wnt/β-catenin also cooperatively promote cell survival under cellular stress conditions. In liver cancer, O-GlcNAcylation of β-catenin promotes cell growth, while also inhibiting the activity of pro-apoptotic factors to protect cancer cells from apoptosis-inducing agents. Increased level of β-catenin is further enhanced by a positive feedback mechanism, where increased β-catenin elevates the expression of UAP1 of the HBP pathway and ultimately increases the level of total O-GlcNAc in liver cancer cells (21). Collectively, these findings reveal significant interactions between OGT/O-GlcNAc expression and the Wnt/β-catenin pathway and their role in regulation of cancer cell growth and survival.

#### OGT/O-GlcNAc regulates Hippo signaling in cancer

In recent years, the role of the Hippo pathway in cancer has drawn increasing attention. Upregulation of the Hippo pathway is often associated with an increase in cell proliferation, apoptosis evasion, tumor growth, and poor patient outcomes (111). Core components of the Hippo pathway are regulated by OGT and O-GlcNAc in cancer. Yes-associated protein (Yap), a key transcriptional activator of the pathway, is directly modified by OGT at Serine residue 109, which promotes the localization of Yap into the nucleus, thus enhancing the transcriptional activity of Yap and the activity of the Hippo signaling (112). OGT also modifies Large tumor suppressor kinase 2 (Lats2), a key kinase of the Hippo pathway, to modulate Lats2-induced phosphorylation of Yap, which contributes to the regulation of the Hippo pathway activity (113). Yap phosphorylation by the Lats1/2 complex is also indirectly regulated by OGT and O-GlcNAc. Under starvation, the global level of O-GlcNAcylation is reduced leading to a decrease in the O-GlcNAcylation level of lipoprotein receptor-related protein 6 (LPR6), which induces phosphorylation of Yap and inhibits Hippo signaling (114). In liver cancer cells, treatment with corosolic acid or inhibition of CDK19, a kinase that regulates proliferation in various cancers, results in reduced O-GlcNAc level and reduced expression of OGT and Yap ultimately leading to impaired cell proliferation as well as defective tumor growth (115). Genetic inhibition of OGT reduces Yap expression in liver cancer cells but also blocks cancer cell proliferation, further highlighting the critical role of OGT and O-GlcNAc in regulating the expression and function of Yap in cancer (115). Interestingly, increases in OGT and O-GlcNAc levels in hepatocarcinoma cells increase the sensitivity of cancer cells to ferroptosis in a Yap-dependent manner. Increased Yap expression in hepatocarcinoma cells leads to an increase in cellular iron concentration needed for ferroptosis. Mutation of the potential O-GlcNAcylation site Threonine residue 241 of Yap abolishes the effect of increased O-GlcNAc in inducing ferroptosis in hepatocarcinoma cells (116). Together, these findings help establish an extensive interconnection between OGT/O-GlcNAc and the Hippo pathway to regulate cancer cell survival and tumor growth.

#### OGT/O-GlcNAc regulates ferroptosis in cancer

Ferroptosis is a form of regulated cell death induced by iron-dependent accumulation of lipid peroxides (117). This cell death mechanism, however, is suppressed in various cancers, mainly through the upregulation of proteins that alleviate oxidative stress and reduce lipid peroxides (118). Multiple proteins involved in ferroptosis or regulation of ferroptosis are regulated by OGT and O-GlcNAc in cancer (119). In hepatocarcinoma, OGT stability is controlled by eukaryotic translation initiation factor 3 subunit H (EIF3H). Inhibition of EIF3H induces ferroptosis, which is reversed by OGT overexpression (120). Inhibition of OGT in osteosarcoma led to increased sensitivity to ferroptosis (121). Pharmacological inhibition of OGT reduces the level of O-GlcNAc and promotes the transportation of ferritin, an iron chelator, to lysosome, thus increasing sensitivity to ferroptosis (121). In liver cancer cells, increased O-GlcNAc levels in combination with overexpression of pro-survival transcription factor c-Jun inhibits ferroptosis induced by erastin, a ferroptosis-inducing compound. Importantly, c-Jun is modified with O-GlcNAc at Serine residue 73, which is essential for the ferroptosis-inhibiting activity of c-Jun, suggesting a role of OGT and O-GlcNAc in suppressing ferroptosis to promote survival of liver cancer cells (122). However, in some other cancers, elevated level of O-GlcNAc is associated with increased ferroptosis. In mesenchymal pancreatic cancer cells, increased O-GlcNAc level induced by high glucose influx leads to ZEB1 O-GlcNAcylation and promotes cancer cell ferroptosis sensitivity (123). Genetic inhibition of OGT partially protects pancreatic cancer cells from ferroptosis induced by RSL3, another ferroptosis-inducing compound (123). In these pancreatic cancer cells, high glucose influx elevates the level of O-GlcNAcylation on ZEB1, a key transcription factor involved in tumor development, at serine residue 555, which promotes ZEB1 stabilization and nuclear localization. Accumulation of ZEB1 in the nucleus drives ferroptosis by inducing the expression of enzymes involved in polyunsaturated fatty acid biosynthesis (123, 124). In hepatocarcinoma cells, increased O-GlcNAcylation level on Yap sensitizes cancer cells to ferroptosis (116). O-GlcNAcylation enhances Yap nuclear localization promoting the expression of transferrin receptor (TFRC), a mediator for iron import that is critical for ferroptosis (116). Together, these recent findings show an emerging role of OGT and O-GlcNAc in regulating ferroptosis and survival (Fig. 2), and targeting the O-GlcNAc cycle in combination with ferroptosis-inducing drugs could be a potential approach to targeting cancer.

#### OGT/O-GlcNAc regulates p53 signaling in cancer

In addition to the major signaling pathways discussed above, cancer cell proliferation and survival are regulated by OGT and O-GlcNAc *via* other factors. The master regulator of cell cycle p53 is directly modified with O-GlcNAc, which protects p53 from degradation in multiple cancers (125). Recent studies have shown that alterations in O-GlcNAc level and OGT expression lead to the activation of p53 in ovarian cancer (126). Overexpression of OGT or increased O-GlcNAc level elevates the stability of p53, promotes p53 nuclear localization, and enhances p53 transcriptional activity (126). The increase in p53 stability and function could be a result of O-GlcNAcylation on p53 at Serine residue 149, which protects p53 from proteasome-dependent degradation (125). OGT can also regulate the activity of other components of the p53 pathway, such as p21. In fibrosarcoma cells, pharmacological or genetic inhibition of OGT increases the expression of p21 impairing cancer cell proliferation. The regulation of p21 by OGT occurs in a p53-dependent manner, where p53 is regulated by OGT to modulate the expression of p21, and in a p53-independent manner, where the stability of p21 is increased by OGT inhibition in p53-deficient cells. Reduced OGT activity prevents the formation of E3-ligase targeting p21 in p53-deficient cancer cells, resulting in cell cycle arrest and reduced proliferation (127).

### OGT/O-GlcNAc regulates drug-resistance mechanisms

#### OGT/O-GlcNAc is involved in apoptosis regulation

The crucial roles of OGT and O-GlcNAc in promoting cancer cell survival *via* regulation of pro-survival factors (45, 68, 128) as described in the previous section suggest that OGT and O-GlcNAc also potentially contribute to promoting resistance to anti-cancer therapeutic agents. Indeed, in breast cancer, increased O-GlcNAc level is associated with Bortezomib resistance. High level of O-GlcNAc leads to the elevated level of O-GlcNAcylation on Forkhead Box A1 (FoxA1) and an increase in FoxA1 stability, which results in the downregulation of pro-apoptotic protein Bcl-2 Interacting Mediator of cell death (Bim) and ultimately promotes Bortezomib resistance. Combined treatment of OGT inhibitor and Bortezomib efficiently induces apoptosis in breast cancer cells (129). Similarly, inhibition of OGT and reduced O-GlcNAc levels in liver cancer synergistically improves the effect of doxorubicin in inducing apoptosis in cancer cells. Combined treatment with OGT inhibitor and Doxorubicin notably increases the percentage of cancer cells undergoing apoptosis, reduces cancer cell proliferation, and impairs tumor growth *in vivo* (130). The same synergetic effect of OGT inhibitor and doxorubicin is also observed in prostate cancer. Treatment with OGT inhibitor shows a remarkable increase in the sensitivity of cancer cells to doxorubicin-induced apoptosis (131). Other reports have also shown the importance of OGT and O-GlcNAc in mediating chemotherapy resistance in cancer. O-GlcNAc modification of Metastasis Associated 1 (MTA1) results in increased resistance to genotoxicity in breast cancer (132). In osteosarcoma, the long non-coding RNA lncEBLN3P, which is involved in various cancers, upregulates the total level of O-GlcNAc to induce resistance to methotrexate in osteosarcoma (17, 132, 133). However, increased O-GlcNAc levels in Bortezomib-resistant lymphoma cells resensitize cancer cells to Bortezomib-induced apoptosis. In these lymphoma cells, high levels of O-GlcNAc and Bortezomib lead to the accumulation of truncated BH3 Interacting Domain Death Agonist (Bid), a pro-apoptotic protein of the Bcl2 family, the level of which is further increased by direct O-GlcNAcylation, and ultimately results in apoptosis (134). These findings support the general role of OGT and O-GlcNAc in promoting and maintaining drug resistance in multiple cancer types.

#### OGT/O-GlcNAc regulates DNA damage repair

Many chemotherapeutic drugs exploit the alteration in DNA damage repair mechanism of cancer cells to induce apoptosis. The DNA damage repair system is extensively regulated by many pathways, including OGT and O-GlcNAc. The levels of OGT and O-GlcNAc increase after cells are exposed to DNA damage-inducing agents, such as radiation or chemotherapeutic drugs (135, 136). A high level of O-GlcNAc leads to the activation of components of the DNA damage repair system, which protects cancer cells from apoptosis. The activation of DNA damage repair kinases such as checkpoint kinase half (Chk1/2) provides a feedback mechanism that further elevates O-GlcNAc level, which further diminishes the effect of anti-cancer treatment (137). In prostate cancer, combined treatment using Cdk9 inhibitor and OGT inhibitor is selectively effective in inducing apoptosis (138). Treatment with Cdk9 inhibitor leads to DNA double-strand breaks, which can be overcome by activating MRE11-mediated DNA damage repair. The loading of MRE11 to chromatin requires previous binding of OGT to the damage site suggesting the essential role of OGT in repairing DNA breaks (139). The critical role of OGT/O-GlcNAc in repairing DNA double-strand break is also observed in breast cancer, where an increase in O-GlcNAc level induces resistance of xenograft tumor against radiation, while inhibition of OGT impairs double-strand break repair and reduces cancer cell growth *in vivo* (140). In osteosarcoma, pharmacological and genetic inhibitions of OGT lead to a reduction in DNA double-strand break repair mediated by RAD52-dependent homology recombination, ultimately resulting in cell cycle arrest and reduced cell proliferation (141).

#### OGT/O-GlcNAc affects the efficacy of immunotherapy

In recent years, the use of immune checkpoint inhibitors in treating cancer has become standard in combating various cancers. However, the efficiency of these inhibitors is still being dampened by various resistance mechanisms (142). OGT and O-GlcNAc have recently been shown to be involved in developing resistance in cancer cells. In liver cancer, pharmacological inhibition of OGT results in a decrease in the level of PD-L1, a key regulator highly expressed in some cancers enabling these cells to evade immune responses. In contrary, increased O-GlcNAc level elevates the level of PD-L1, suggesting a potential regulation of PD-L1 by OGT and O-GlcNAc. The stability of PD-L1 in cancer cells increases with elevated levels of O-GlcNAc and decreases with the reduction in the level of O-GlcNAc, suggesting that OGT/O-GlcNAc regulates PD-L1 stability. This regulation is independent of the proteasome but relies on lysosomal degradation. O-GlcNAc inhibits the sorting of PD-L1 to lysosomes protecting PD-L1 from degradation and promoting immune evasion of cancer cells (143). In pancreatic cancer, the level of O-GlcNAc is positively correlated with the level of PD-L1 and increases the tumorigenicity of cancer cells. Increased O-GlcNAc level in pancreatic cancer leads to increased O-GlcNAcylation of Myc, which in turn elevates the transcription of PD-L1, resulting in an increase in immune evasion of cancer cells (144). In esophageal carcinoma, the expression of OGT is elevated and is associated with the immunosuppression activity of the tumor. Exosomes derived from esophageal carcinoma cancer stem cells containing OGT target cytotoxic CD8+ cells, elevating the expression of PD-1, the receptor for PD-L1, which leads to suppression of immune activity, thus protecting cancer cells from the cytotoxic activity of the immune system (145).

Together, these findings reveal a significant contribution of OGT and O-GlcNAc in promoting cancer cell survival during chemo- and immunotherapy and contributes to avoiding immune destruction (Fig. 2). Therefore, targeting OGT and O-GlcNAc in combination with current treatments may have potential in improving anti-cancer therapy.

### Role of OGT in metastasis

The progression of cancer is characterized by tumor growth, invasion, and the ability of cancer cells to escape primary tumors and metastasize to distant organs. During the process of metastasis, cancer cells invade nearby tissue, break through the extracellular matrix and intravasate into blood vessels. Once in the bloodstream, cancer cells travel through the body and colonize at distant tissues, growing as metastases (146). During this process, cancer cells are extensively regulated by multiple factors, from genome instability to signal transduction, as well as by stimuli from the tumor microenvironment (146). OGT and O-GlcNAc can significantly contribute to modulating the metastasis potential of cancer cells. Expression of OGT and O-GlcNAc levels are highly elevated in metastasized thyroid tumors when compared to primary tumor. Elevated levels of OGT and O-GlcNAc in thyroid cancer cells are also associated with increased invasion and migration capacity of cancer cells. Inhibition of OGT in thyroid cancer cells notably reduces invasion, migration, and metastasis ability of thyroid cancer cells (112). Similarly, O-GlcNAc level in breast cancer tissue is higher compared to nearby tissue and is higher in metastatic lymph nodes when compared to primary tumors (147). Genetic inhibition of OGT in breast cancer cells significantly reduces metastasis in mice models (87, 147). Inhibition of OGT in prostate cancer cells also remarkably reduces metastasis incidents in mice models (148).

OGT and O-GlcNAc also modulate the expression and activity of various genes and proteins controlling the process of metastasis. In hepatocarcinoma, O-GlcNAcylation of lysine acetyltransferase 5 (KAT5) is critical for cancer cell migration, invasion, and metastasis. Inhibition of OGT or reduced expression of KAT5 impaired invasion ability and lung metastasis of hepatocarcinoma cells (149). In addition, OGT also directly modifies Rab3A in hepatocarcinoma cells to regulate the metastasis ability of these cancer cells. Rab3A is a Ras-like GTPase that is upregulated in hepatocarcinoma tissues but is inversely correlated with invasion and metastasis of liver cancer cells. O-GlcNAcylation of Rab3A inhibits the GTP-binding activity of Rab3A, thus impairing the function of Rab3A in suppressing metastasis. Increased OGT expression in hepatocarcinoma cells completely abolishes the negative effect of Rab3A overexpression in promoting lung metastasis *in vivo* (150). In non-small lung cancer cells, increased O-GlcNAc levels enhanced the migration ability of cancer cells. In these cancer cells, O-GlcNAc is regulated by Transient receptor potential melastatin 7 (TRPM7), a cation channel that is highly expressed in cancer. TRPM7 regulation of O-GlcNAc modulates the expression of c-Myc and caveolin-1, which in turn regulate the metastasis ability of lung cancer cells. Inhibition of TRPM7 reduces OGT expression and decreases the level of O-GlcNAc, and subsequently the level of c-Myc and metastasis in mice models (151). In lung cancer cells, OGT also regulates metastasis ability *via* activating IL-6/STAT3 signaling. OGT directly modifies STAT3, which enhances phosphorylation of STAT3 to promote metastasis in lung cancer (152). In colorectal cancer, levels of OGT and O-GlcNAc are significantly higher in lymph node metastases when compared to primary tumors. Increased OGT expression enhances EZH2 stability *via* direct O-GlcNAcylation, thus promoting cancer cell migration and invasion (153). Increased O-GlcNAc level in colorectal cancer cell also increases the expression of β-catenin, which in turn promotes metastasis *in vivo* (106).

The regulation of metastasis by OGT and O-GlcNAc occurs not only in cancer cells but also in other cells that contribute to the tumor microenvironment. In tumor-associated macrophages (TAM), the level of O-GlcNAc is highly elevated in tumor-promoting M2-macrophage-like TAMs, partly as a result of increased glucose uptake by macrophages. Increased O-GlcNAc level in M2-macrophage-like TAMs enhances metastasis and increases drug resistance of cancer cells. In the M2-macrophage-like TAMs, OGT directly modifies protease Cathepsin-B at Serine residue 210, which promotes maturation and secretion of Cathepsin-B to the tumor microenvironment to degrade the extracellular matrix supporting metastasis activities of cancer cells. Inhibition of Cathepsin-B O-GlcNAcylation impairs metastasis of cancer cells, indicating a critical role of OGT and O-GlcNAc in remodeling the tumor microenvironment to support invasion and metastasis (154).

These results highlight the direct involvement of OGT and O-GlcNAc in regulating metastasis, potentially by regulating the ability of cancer cells to acquire migration and invasion abilities to form metastatic colonies at distant organs.

### OGT/O-GlcNAc regulates EMT

The transition of cancer cells during metastasis involves complex alterations in gene expression profiles, signaling transduction, and metabolism. During this process, cancer cells lose epithelial traits while gaining mesenchymal characteristics, including increased invasion, migration, stem-like cell properties, and resistance to therapy. This transition process of cancer cells is called EMT (155, 156). The process of EMT is regulated by a set of core transcription factors (157), as well as major signaling pathways, including OGT and O-GlcNAc (158).

The process of EMT in multiple cancers is promoted by OGT and O-GlcNAc. In endometrial cancer cells, increased O-GlcNAc level induces EMT, which is indicated by increased expression of pro-EMT genes, as well as cytoskeleton reorganization and increased cellular motility (159). Similarly, increased O-GlcNAc level induced by an increase in the HBP activity in non-small lung cancer cells also drives cancer cells to undergo EMT, resulting in elevated invasion and migration (160). In breast cancer, increased OGT expression results in increased levels of pro-EMT proteins, such as vimentin and fibronectin, while genetic inhibition of OGT decreases the expression of these pro-EMT factors (161). Recently, quantitative imaging analysis of O-GlcNAc level in tumor tissues using mass spectrometry revealed a remarkably higher level of O-GlcNAc at the invasive front of the tumor. The invasive front of a tumor is the place where cancer cells are actively undergoing EMT, which further implicates the crucial role of OGT and O-GlcNAc in regulating EMT (162).

The process of EMT is regulated by a set of transcription factors, including proteins of the Snail family (163), ZEB family (164), and the TWIST family (165). The expression and activity of these transcription factors are regulated by OGT and O-GlcNAc. Snail is directly modified by OGT at Serine residue 112. This modification competes with phosphorylation on Snail and protects the protein from degradation elevating the transcriptional activity of Snail to drive EMT (166). In addition, OGT and O-GlcNAc also regulate the expression and function of E-cadherin, a marker of epithelial cells, the expression of which declines as the first hallmark of EMT. Increased O-GlcNAc level reduces the level of E-cadherin in colorectal cancer cells (153), while inhibition of OGT results in an increased level of E-cadherin (153, 167). OGT also modifies the intracellular domain of E-cadherin to prevent the recycling of E-cadherin, thus reducing the expression of E-cadherin on cell membranes and promoting EMT (168). O-GlcNAc modification of E-cadherin and its binding partner, β-catenin, also disrupts the membrane complex of E-cadherin/β-catenin, allowing β-catenin to translocate to the nucleus and activate expression of pro-EMT genes (106, 169).

The process of EMT is extensively modulated by other signaling pathways that are under the control of OGT and O-GlcNAc. One such pathway is the hypoxia response pathway. The transcription factor HIF-1α, which is stabilized under hypoxic conditions, is protected from degradation by OGT and O-GlcNAc in breast cancer cells even under normoxia. Increased OGT and O-GlcNAc levels in these cells reduce the level of alpha-ketoglutarate and block hydroxylation of HIF-1α stabilizing HIF-1α under normal oxygen tension (170). Together, increased HIF1-α level elevates the expression of pro-EMT factors (171). In addition, the expression of E-cadherin is directly suppressed by the transcription factor kruppel-like factor 8 (KLF8) (172, 173). KLF8 expression is positively correlated with OGT and O-GlcNAc levels in breast cancer cells and is regulated by OGT at both the protein and mRNA levels, suggesting a potential regulation of EMT by OGT *via* KLF8 (172, 173, 174). However, in hepatocarcinoma cells, a high level of O-GlcNAc is critical to the maintenance of high levels of expression of EMT negative regulator FOXO1, suggesting a more complicated regulation of EMT by OGT and O-GlcNAc, which is not limited only to promoting EMT, but also in maintaining a hybrid state of the EMT, needed for metastasis (175, 176). Thus, OGT and O-GlcNAc play a positive role in regulation invasion and metastasis (Fig. 2) in a number of cancers.

### OGT/O-GlcNAc regulates cancer cell plasticity and stem-like cell properties

One of the major drivers of tumor metastasis is a subpopulation of cancer cells that possess the capacity to cell renew and differentiate known as cancer stem-like cells (177). The self-renewing and differentiating capacity of cancer stem-like cells are believed to be critical during the growth of metastases at distant organs. For example, the major population of circulating cancer cells during metastasis in colorectal cancer are negative for Lgr5, a cancer stem-like cell marker, or Lgr5-non-cancer stem cells. However, during the development of micro- and macro-metastases, the Lgr5+ cells are indispensable (178). In addition, during the process of EMT, which is critical for metastasis, cancer cells not only gain increased motility, invasion, and migration capacity but also gain stem-like cells traits (177). Importantly, the EMT of cancer cells occurs through distinct cancer cell states, and the hybrid state of EMT shows the highest metastasis capacity (155). Interestingly, recent studies have pointed out that the hybrid state of EMT also showed the highest stem-like cell potential (179, 180), suggesting an indispensable connection between cancer stem-like cells and metastasis.

Recent studies have started to connect the regulation of cancer stem-like cells to OGT and O-GlcNAc. Elevated levels of OGT and O-GlcNAc in breast cancer led to an increase in cancer stem-like cell populations, as well as the tumor initiation capacity of breast cancer cells (161). Genetic or pharmacological inhibition of OGT decreases cancer stem-like cell populations as well as the expression of transcription factors involved in promoting self-renewing capacity (161). Similarly, increased O-GlcNAcylation by IL-8 stimulation in colon and lung cancer cells results in increased cancer stem-like cell populations, which can be inhibited by treatment with an OGT inhibitor (181). Inhibition of OGT also leads to reduced cancer stem-like cell populations and self-renewing capacity in leukemia, potentially *via* regulation of STAT3/5 signaling (182). A high O-GlcNAc level is also required for maintaining cancer stem-like cell generation and the expression of stem cell factor SOX2 (183). OGT and O-GlcNAc are also critical for the expression of cancer stem-like cell markers, such as CD44 and CD133 in colon cancer (184).

In addition to directly regulating cancer stem-like cell properties, OGT and O-GlcNAc also modulate the expression and activity of other factors to induce stem-like cell traits in cancer. KLF8 is a driver of EMT in breast cancer (172) and its expression in breast cancer cells is critical to the maintenance of breast cancer stem-like cells, as well as the expression of stem cells factors (174). Importantly, KLF8 is required for OGT to promote cancer stem-like cells phenotype in breast cancer, and KLF8 and OGT form a feed-forward loop to further induce stem-like cells traits in cancer cells (174). Cancer stem-like cell population in colon cancer is negatively regulated by MYBL1, which is a target for O-GlcNAc regulation. In colon cancer cells, O-GlcNAc epigenetically regulates the expression of MYBL1, a protein that is highly expressed in cancer to promote malignant development, which in turn regulates the population of cancer stem-like cells and tumor growth (185). In addition, many stem cell factors are directly O-GlcNAcylated, which could potentially contribute to cancer stem-like cell regulation by OGT/O-GlcNAc. C-Myc is a stem cell factor that is highly expressed in many cancers and is associated with stem-like properties (186, 187). C-Myc is directly O-GlcNAcylated by OGT, which enhances its stability and increases the activity of this oncoprotein (188). SOX2, another stem cell factor, is also directly modified by OGT in pancreatic cancer cells, which protects SOX2 from degradation and increases the self-renewing capacity of pancreatic cancer cells (189). During EMT, cancer cells also gain stem-like cells phenotypes. The expression and activity of EMT regulators are extensively regulated by OGT and O-GlcNAc as discussed above. Together, these findings begin to reveal the indispensable role of OGT and O-GlcNAc in regulating cancer stem-like cells and phenotypic plasticity (Fig. 2), which may also contribute to the regulation of cancer metastasis.

Overall, increased OGT and O-GlcNAc levels in cancer cells potentially govern a spectrum of cancer-promoting activities through mechanisms (Fig. 2) that may be cancer-cell or context-specific.

## Targeting OGT and O-GlcNAc

Previous discussion has highlighted the crucial role of OGT and O-GlcNAc during tumor progression and the potential for OGT/O-GlcNAc as a therapeutic target in cancer treatment. However, since OGT is an essential protein in adult mice (190), targeting OGT could have unwanted side effects (191). Nevertheless, various selective inhibitors of OGT have been developed and tested in cancer cells. Inhibitors selectively targeting OGT include substrate analogs, bisubstrate inhibitors, and small molecule inhibitors (192). However, the application of these inhibitors is greatly hindered by various challenges, including poor permeability, possible toxicity, and lack of target specificity (192). For example, some of the first introduced inhibitors of OGT, which compete for the substrate binding sites include UDP-sugars, are UDP-S-GlcNAc or 5SGlcNHex (193, 194). These inhibitors initially showed promising specificity in targeting OGT, but UDP-S-GlcNAc and similar compounds were eventually discovered to have poor cell permeability resulting in an undesirable inhibitory effect (192). 5SGlcNHex is a newly developed compound that is balanced in hydrophobicity and hydrophilicity, a characteristic that enables 5SGlcNHex to enter the cells, where it is metabolized to generate UDP-5SGlcNHex to inhibit the process of O-GlcNAcylation, including *in vivo* (193). However, it is possible that the inhibitory effect of UDP-5SGlcNHex may alter the function of other glycosyltransferases, resulting in a lack of specificity and thus potential unwanted side effects (192).

Another widely used small molecule inhibitor of OGT is OSMi-1, which is cell permeable and shows high inhibitory and specificity toward OGT (195). However, the use of OSMi-1 may lead to a reduction in cell viability, which is possibly due to an unwanted side effect (192). Based on OSMi-1, other newly developed inhibitors are also available, such as OSMi-4. OSMi-4 shows nanomolar affinity to OGT and selectively inhibits the purified enzyme (196). However, the specificity of OSMi-4 in cells and *in vivo* still requires extensive investigation. Together, these challenges have slowed the testing of OGT inhibitors in preclinical cancer models.

It is likely that OGT and O-GlcNAc play a critical role in maintaining homeostasis in non-cancer cells, and thus targeting OGT with small molecule inhibitors could result in unwanted side effects. Nevertheless, the potential of using OGT inhibitors in treating cancer should be explored. Targeting OGT may have clinical utility, as seen with other inhibitors that target broad sets of proteins, including HDAC or proteasome inhibitors that are currently used in the clinic to treat cancer (197, 198). OGT inhibitors can potentially be used in combination with other chemotherapeutic or targeted therapies to enhance the sensitivity of cancer cells to anti-cancer treatment. A recent study in hepatocarcinoma showed an increase in the sensitivity of cancer cells to doxorubicin, a widely used DNA-damage-inducing drug, when the level of O-GlcNAc was reduced (199). Similarly, the combination use of OSMi-1 and doxorubicin induces a greater extent of apoptosis and tumor growth *in vivo* when compared to each single drug (130). In prostate cancer, genetic and pharmacological inhibitions of OGT sensitize the cells to apoptosis induced by docetaxel, an anti-cancer drug that inhibits depolymerization of microtubules and cell division (200). The development of an OGT pro-drug inhibitor that specifically delivers high doses of the drug to tumor tissue while sparing normal tissue could have clinical benefits. This approach has been successful with 6-Diazo-5-oxo-l-norleucine (DON), which targets several glutamine-using enzymes (201) and these pro-drugs are currently in clinical trials for cancer treatment (https://classic.clinicaltrials.gov/ct2/show/NCT04471415). Together, these findings support the potential of using an OGT inhibitor in combination with other therapeutic agents to enhance the efficacy of current cancer treatments. However, there is still a dire need to develop novel inhibitors targeting OGT and O-GlcNAc with greater permeability, specificity, and stability to test these ideas further.

## Conclusion and future perspectives

In conclusion, OGT and O-GlcNAc play a critical role during tumor progression. Recent studies have further highlighted the involvement of OGT and O-GlcNAc in regulating various oncogenic events during tumor development that drive cancer cell proliferation, survival, plasticity, and metastasis. The roles of OGT and O-GlcNAc in regulating key events of tumor progression make OGT a potential therapeutic target for cancer treatment. The use of OGT inhibitors in recent reports has shown a potential in targeting OGT in combination with other drugs to reduce tumor growth and induce apoptosis in cancer cells. However, the application of OGT inhibitors is still limited due to various challenges, such as a lack of stable and specific inhibitors, possible toxicities, and unwanted side effects. Moreover, there exist few stable and specific OGT inhibitors that are available to test in preclinical cancer models and the pharmacological effect of OGT inhibitors *in vivo* is still understudied. Thus, more specific, novel OGT inhibitors are needed to test the potential of targeting OGT in treating cancer.

## Conflict of interest

The authors declare that they have no conflicts of interest with the contents of this article.
